# Transdermal Delivery of Baicalin Based on Bio‐Vesicles and Its Efficacy in Antiaging of the Skin

**DOI:** 10.1111/jocd.70024

**Published:** 2025-02-13

**Authors:** Liang Chen, Fudi Wang, Xiaoyun Hu, Nihong Li, Ying Gao, Fengfeng Xue, Ling Xie, Min Xie

**Affiliations:** ^1^ Scientific Research Laboratory Shanghai Le‐Surely Biotechnology Co. Ltd Shanghai China; ^2^ SASELOMO Research Institute and Biological Laboratory Shanghai Chuanmei Industrial Co. Ltd Shanghai China; ^3^ Evelab Insight (Singapore) Pte. Ltd Singapore Singapore; ^4^ Zhejiang Moda Biotech Co. Ltd Hangzhou China; ^5^ Nanomedicine and Intestinal Microecology Research Center, Shanghai Tenth People's Hospital, School of Medicine Tongji University Shanghai China

**Keywords:** antiaging, baicalin, bio‐vesicle, skin physiology and cell culture, transdermal delivery

## Abstract

**Objective:**

To develop a stable and efficient delivery system for baicalin, a flavonoid with potential antioxidant and antiaging properties, to overcome its limitations in solubility, stability, and skin permeability.

**Methods:**

Baicalin was encapsulated using ATP synthase molecular motor technology into bio‐vesicles derived from yeast/bacillus cell membranes, forming “motor baicalin” (MB). The liposome baicalin (LB), baicalin raw material (BRM), and bio‐vesicles were used for comparison. The stability, transdermal penetration, and antioxidant activity of MB, LB, BRM, and bio‐vesicles were evaluated through in vitro and in vivo tests.

**Results:**

MB formed a stable core‐shell structure, significantly enhancing the water solubility and long‐term stability of baicalin. The tests confirmed superior transdermal penetration and antioxidant activity of MB, evidenced by increased expression of SOD, CAT, and GSH‐Px enzymes and improved cell proliferation and migration. Clinical trials demonstrated significant reductions in wrinkle depth and improvements in skin elasticity.

**Conclusion:**

This study presents a promising approach to improving the stability and transdermal delivery of baicalin. MB showcases potent antioxidant and antiaging properties, making it a valuable component in skincare products.

## 1 Introduction

Skin aging, characterized by increased wrinkles, dullness, dryness, pigmentation, and loss of elasticity, is a complex biological process induced by intrinsic and extrinsic factors over time [1]. Generally, UV radiation, inflammation, glycation, and reactive oxygen species (ROS) are believed to be the primary contributors to skin aging [2]. Among these, ROS is considered to play a dominant role, as the other three factors ultimately accelerate skin aging by upregulating ROS levels in the skin [3, 4]. Although it is well known that low concentrations of ROS have positive effects on cell signaling, cell proliferation, differentiation, and autophagy [5], excessive ROS accumulated over the years can attack cells and tissues, leading to the loss of collagen and intercellular lipids and inducing skin aging. Consequently, understanding the relationships between ROS and skin aging phenotypes, as well as developing new and highly effective antioxidants are current research hotspots in cosmetic industry [6, 7, 8].

In recent years, numerous antioxidant ingredients have been developed and incorporated into cosmetics, such as Vitamin C (VC), Vitamin E (VE), ferulic acid, coenzyme Q10, and resveratrol. However, the effectiveness of these antioxidants is limited by their structural instability and low antioxidant efficiency [9, 10, 11]. As is well known, there are two levels of antioxidant systems in our skin: relatively inefficient nonenzymatic defenses and highly efficient enzymatic defenses. The nonenzymatic antioxidant system neutralizes ROS through compounds like VC, VE, and certain carotenoids, whereas the enzymatic antioxidant system primarily relies on activating the nuclear factor erythroid 2‐related factor 2/antioxidant response element, (Nrf2/ARE) signaling pathway. This activation promotes the expression of powerful antioxidant enzymes, such as catalase (CAT), superoxide dismutase (SOD), and glutathione peroxidase (GSH‐Px) [12]. Therefore, developing more advanced antioxidant ingredients necessitates finding a substance that activates and stabilizes the Nrf2/ARE pathway.

Baicalin, a flavonoid derived from Scutellaria baicalensis Georgi, exhibits a range of biological activities including anti‐inflammatory, antineoplastic, and immunoregulatory properties. It has, thus, become a significant pharmacological component in the biomedical field [13, 14]. Recent studies have demonstrated that baicalin possesses excellent antioxidant properties by activating the Nrf2/ARE pathway, making it a promising antioxidant for use in cosmetics [15]. However, baicalin monomer is insoluble in water and polyol solvent, making it prone to precipitation from formulations, which results in product instability. Furthermore, the precipitated baicalin is susceptible to discoloration and off‐odor under high temperature and light conditions. This not only compromises the efficacy of the products but also significantly affects the user experience. Moreover, BRM has been shown to have poor skin absorption, potentially leading to excessive local epidermal concentration and skin irritation. Therefore, in order to expand the application of baicalin in cosmetics, it is essential to address these aforementioned drawbacks first. In fact, recent advancements in transdermal drug delivery systems used in cosmetics can effectively resolve similar issues. For instance, liposome encapsulation technology not only stabilizes certain ingredients but also enhances their transdermal absorption, thereby improving the efficacy while reducing irritation.

In this study, we developed a novel baicalin delivery system based on our previous advancements in ATP synthase molecular motor technology [16, 17]. The ATP molecular motor, also known as ATP synthase, plays a crucial role in cellular energy metabolism, encompassing processes such as nerve conduction, muscle contraction, and substance transport. This molecular motor features a distinctive bio‐vesicle structure that can encapsulate active molecules for effective delivery. Our previous research has demonstrated that the ATP molecular motor facilitates directional movement of the entire loading system with F1 rotating during ATP hydrolysis [18]. Leveraging the low pH active targeting capability of ATP molecular motors and the weak acidity characteristic of human skin tissue, we found that baicalin encapsulated within these bio‐vesicles can effectively penetrate deep into the skin. This mechanism is essential for enhancing the transdermal delivery efficiency of this technology.

Specifically, BRM is encapsulated within bio‐vesicles to create a “core‐shell” structure. The resulting baicalin delivery system is designated as “motor baicalin” (MB). For comparison, the baicalin raw material (BRM), bio‐vesicles (without baicalin), and liposome baicalin (named LB, a commonly used modified technology in cosmetics) also proceeded. As a result, both in vitro and in vivo tests have shown that MB exhibits excellent transdermal properties along with antiaging and skin‐repairing effects. This study presents a promising strategy to address challenges related to instability and transdermal delivery of active ingredients in cosmetic formulations.

## 2 Materials and Methods

### 2.1 Materials

Commercial baicalin (Cosmetic Grade; LogP: 0.1422; Solubility: nearly insoluble in water and polyol solvent; Stability: stable in dilute acid) was purchased from Shanxi Jiahe Biotechnology Co. Ltd. Retinol powder (≥ 95%) was obtained from Sigma‐Aldrich Reagent Company. Trifluoroacetyl tripeptide‐2 powder was sourced from Shanghai Peptide Biotechnology Co. Ltd., PR China. Acetone (AR), isotonic phosphate buffer, and fluorescein (AR) were acquired from Shanghai Titan Technology Co. Ltd. NaCl (AR), propylene glycol (AR), hydrogenated lecithin (AR), caprylic acid glyceride (AR), disodium EDTA (AR), DAPI solution, and other reagents were purchased from Greagent Company. SOD test kit, CAT test kit, GSH‐Px test kit, hematoxylin, Ponceau‐Fuchsin, and aniline blue were purchased from Beyotime Biotechnology. Purified water (18 MΩ) was obtained using a Milli‐Q system (Millipore). All the chemical reagents were utilized without further purification.

### 2.2 Instruments

The MB or LB sample was sonicated by the bath type of sonication (KQ‐700DM) from Kunshan Ultrasonic Instrument Co. Ltd. (China) with the ultrasonic frequency of 40 KHz at 25°C. In these sonication conditions, no structural modifications occurred during the sonication process. DLS and zeta potential were measured with a Malvern Nano‐ZS90 (Britain). Transmission electron microscopy (TEM) was performed using an Ultra 55 and an FEI Tecnai 20 operating (America) at 200 KV. The test sample was dispersed in an ethanol/water mixture (7:3, v/v), sonicated for 5 min, and then dropped onto a copper grid for examination. HPLC measurements were conducted on a 1260 Infinity system (Agilent, America) with Elite C18 OSD‐BP column. The confocal images were measured by a Nikon single‐photon confocal microscope (NikonC1Si, Japan). The fluorescence spectra were conducted on a fluorescence microscope (Leica Microsystems Model DMi8 manual, Germany). Stability was tested in an incubator with different temperatures (YiHeng Shanghai, DHP9402). pH values were measured by a pH meter (Mettler Toledo, S400‐K).

### 2.3 The Preparation of MB

The preparation of MB was conducted following a previously reported method with appropriate modifications, and the utilizing bio‐vesicles derived from the cell membranes of yeast and *Bacillus* [18]. The detailed procedure is outlined as follows:

#### 2.3.1 Preparation of Baicalin Cyclodextrin Encapsulated Nanoparticles

Cyclodextrin (5 equal) is weighed and subjected to grinding in a colloid mill. Subsequently, baicalin (15 equal) is also weighed and added to the colloid mill. The mixture is thoroughly ground to ensure homogeneity. Following this, water (100 equal) is introduced into the mixture to completely dissolve the evenly mixed cyclodextrin–baicalin powder. The resulting solution undergoes homogenization using a high‐pressure microjet homogenizer at a pressure of 30 MPa. This homogenization process is repeated several times to achieve uniformity. Finally, the solution is collected and subjected to freeze‐drying at −80°C to remove the solvent, achieving baicalin cyclodextrin encapsulated nanoparticles.

#### 2.3.2 Cultivation of *Bacillus subtilis* and Purification of the Vesicle Fraction

The freeze‐dried *Bacillus subtilis* powder is dissolved in nutrient broth (NB) medium. The resultant bacterial suspension is transferred into sterile test tubes containing an appropriate volume of liquid culture medium and mixed thoroughly for cultivation purposes. Subsequently, the activated bacterial strain is introduced into a conical flask filled with NB medium and incubated under optimal shaking conditions for further use. Afterward, *Bacillus subtilis* cells (24 equal) are transferred into NB liquid medium supplemented with a designated amount of yeast powder (55 equal) for further cultivation. Upon completion of this cultivation phase, the fermentation broth is collected and centrifuged to obtain bacterial sediment.

The bacterial sediment is then resuspended in a phosphate buffer solution at a defined volume ratio. This buffer solution containing the bacterial cells undergoes homogenization followed by treatment with high‐pressure microjet homogenization at a pressure of 30 MPa. The resultant solution containing bacterial fragments is passed through a solid chromatography column to separate and purify the vesicle fraction.

#### 2.3.3 Preparation of the Target MB Product

A measured quantity of the vesicle fraction is diluted with phosphate buffer solution. An appropriate amount of baicalin cyclodextrin encapsulated nanoparticles is weighed and combined with the vesicle fraction. The mixture undergoes ultrasonic treatment using an ultrasonic instrument to ensure thorough mixing. The resulting liquid is then transferred to a high‐pressure extruder, where nitrogen serves as the pressure source. The mixture is extruded through a polycarbonate membrane and collected via centrifugation. Following precipitation, the product is dried and ground to obtain the final target product.

### 2.4 The Preparation of LB

A specific amount of propylene glycol (35.5%) and hydrogenated lecithin (7%) are weighed and stirred for 10 min at 75°C and 600 rpm until all particles dissolve in the liquid. Subsequently, an appropriate quantity of caprylic acid glyceride (4.5%) is added to this solution, which continues stirring for an additional 5 min under identical conditions until a homogeneous mixture is achieved.

Next, a measured amount of baicalin (8.5%) is introduced into the mixture, followed by stirring for another 10 min at 75°C and 600 rpm until a uniform yellow liquid forms. Afterward, an appropriate amount of disodium EDTA (0.05%) is incorporated into the solution and stirred for 5 min at the same temperature until achieving a consistent yellow liquid.

The resultant solution undergoes homogenization using a microjet homogenizer across three cycles at a pressure of 2000 psi while maintaining discharge temperatures at 50°C during homogenization and cooling down to 15°C afterward. This process yields a slightly viscous yellow liquid. Finally, the solution centrifuged at 5000 rpm for 10 min to eliminate any insoluble solid impurities.

### 2.5 Stability Test

The structure stability of MB was assessed by monitoring changes of zeta potential and particle size over a period of 90 days across temperatures: −20^o^C, 4^o^C, room temperature (RT), and 40^o^C through a Malvern Nano‐ZS90. We also monitored the change of loading capacity and pH value within 90 days at different temperatures of −20^o^C, 4 ^o^C, RT, and 40^o^C by using HPLC and S400‐K.

### 2.6 Detection of Baicalin Loading Capacity in MB

The baicalin loading capacity was determined using high‐performance liquid chromatography (HPLC), with a baicalin standard sample sourced from Aladdin, which has a purity of ≥ 98%. The conditions and samples used were as follows: an Elite C18 OSD‐BP column maintained at 25°C, a flow rate of 1 mL/min, and a detection wavelength set at 280 nm. The mobile phase consisted of methanol and 0.2% of phosphoric acid in a ratio of 47:53. Acetone was used as the solvent for the standard curve samples, while pure water was used for the test samples. The calculation of standard curve equations (concentration range from 0 to 100 μg/mL) was *y* = 34.526x‐28.428 with the *R* ^2^ value of 0.9987 (Figure S1).

### 2.7 In Vitro Transdermal Release Experiment (Franz Diffusing Cells)

The test was conducted at 32°C using an isotonic phosphate buffer (pH 7.4) as the medium. When the amount of baicalin added was 2 mg/mL, the solution became clear and transparent, and no precipitation or stratification was observed after being stored at room temperature for 48 h. Three groups were established: the BRM group, the LB group, and the MB group, each with three parallel subgroups. The skin samples were obtained from the back of 1 kg New Zealand white rabbits (the related animal study was approved by the Ethics Committee on Institute of Basic Medicine and Cancer, Chinese Academy of Sciences with the approval number 2023R0071). Following isoflurane respiratory anesthesia, the rabbits were humanely euthanized by injecting 1 mL of an appropriately concentrated potassium chloride solution. The back skin was harvested, with the hair and fat layers removed, thoroughly washed with water, divided into appropriate sizes, and stored in normal saline at 4°C for later use. The active solutions used in the diffusion cell were diluted with isotonic buffer. The concentrations of BRM, MB, and LB were 150 μg/mL, 1 mg/mL, and 1.76 mg/mL, respectively (both equivalent to 150 μg/mL of BRM). For each experiment, 2 mL of the active solution was added to the donor cell. The receiving cell contained 8 mL of isotonic phosphate buffer (potassium chloride, sodium chloride, pH 7.4). The skin was placed between the donor and receiving cells and clamped in the transdermal instrument. At specified time intervals, 0.5 mL of the receiving cell solution was withdrawn and replaced with an equal volume of isotonic solution. After collecting samples at each time point, the solutions were analyzed using a UV spectrophotometer at 280 nm. The concentration of baicalin was calculated using a baicalin standard curve. The transmittance was then calculated to create the chart.

### 2.8 Transdermal Penetration Test

The baicalin molecule itself lacks fluorescence, rendering it unsuitable for tracking via fluorescence methods. Therefore, in this study, baicalin was labeled with fluorescein isothiocyanate (FITC) to facilitate fluorescence tracking. The labeling procedure is outlined as follows (Figure S2): Appropriate amounts of baicalin were dissolved in an acetic acid–sodium acetate buffer. Subsequently, tyramine (p‐hydroxyphenethylamine) and sodium cyanoboride were added successively, the mixture was then stirred in a water bath under dark conditions. FITC was introduced to the tyraminated baicalin using borate buffer as the labeling medium and allowed to react overnight. Denaturing vertical plate polyacrylamide gel electrophoresis was employed subsequently to detect and separate the FITC‐labeled baicalin. Further purification involved separating the free baicalin from the labeled compound using a glucose gel column, followed by elution with Tris–HCl to obtain FITC‐labeled baicalin.

The experiment utilized skin from 3‐month‐old miniature pigs. Following hair removal, a scraper was used to remove the fat layer and a portion of the dermis. The skin was then cut into circular pieces, each approximately 3.14 cm^2^ in area. These cuticles were secured on the penetration device with the epidermis facing upward.

The MB actives, LB actives, and BRM sample were diluted to a concentration of 2‰ (2 mg/mL, based on the baicalin concentration) with PBS buffer at pH 7.4. Subsequently, 0.5 mL of each diluent was added to the penetration device and incubated in a dark environment for 1 h. Postincubation, the skin was removed and meticulously rinsed with water until no surface fluorescence discoloration was observed. The surface water was absorbed using absorbent paper, and the skin was slightly dried in the dark. The samples were then sectioned and observed under a fluorescence microscope to assess the penetration of fluorescence into the skin.

### 2.9 Cell Uptake Assay

L929 fibroblasts with a density of 1 × 10^6^ cells per well were seeded in dishes overnight and subsequently incubated with FITC‐labeled BRM, FITC‐labeled MB, and FITC‐labeled LB at equivalent concentrations for 2, 4, and 8 h and then, stained with DAPI (P0131, BYT) at room temperature. The cellular uptake and intracellular distribution were observed by a Nikon single‐photon confocal microscope. The FITC‐labeled baicalin can reflect the “uptake effect” of cells on baicalin by observing the intracellular fluorescence intensity at specific excitation/emission wavelengths. Fluorescence intensity was measured using a fluorescence enzyme‐linked immunosorbent assay (ELISA) reader, and semiquantitative analysis of optical density intensity was performed using Image J.

### 2.10 Anti‐ROS Property

The anti‐ROS detection was conducted using a 2′, 7′‐dichloro‐fluorescein diacetate (DCFH‐DA) stain assay. The experiment was divided into six groups: Control, UVB or H_2_O_2_, MB (133.3 μg/mL, equal with BRM 20 μg/mL), LB (235 μg/mL, equal with BRM 20 μg/mL), BRM (20 μg/mL), and bio‐vesicles (113.3 μg/mL, equal with MB without BRM). Human skin fibroblast (HSF) cells were plated in 12‐well plates (2 × 10^5^ cells per well) and treated with cell culture medium, MB, LB, BRM, and bio‐vesicles for 24 h. The cells were then exposed to UVB (200 mJ/cm^2^) or H_2_O_2_ (1.6 mM, for 30 min) to induce ROS except for the control group. Subsequently, the cells were incubated with 10 μM DCFH‐DA for 20 min at 37°C. After washing with PBS, fluorescence images were visualized using a fluorescence microscope (Leica Microsystems Model DMi8 manual).

### 2.11 The Enzymatic Activities of SOD, CAT, GSH‐Px In Vitro

The enzymatic activities of SOD, CAT, and GSH‐Px were tested in HSF cell lines. Hydrogen peroxide (H_2_O_2_) and UVB radiation were introduced as stimuli to establish a ROS damage model. The experiment was divided into six groups: Control, UVB or H_2_O_2_, MB (133.3 μg/mL), LB (235 μg/mL), BRM (20 μg/mL), and bio‐vesicles (113.3 μg/mL). HSF cells were seeded into a six‐well plate and incubated with the cell culture medium, MB, LB, BRM, and bio‐vesicles for 24 h. Subsequently, the cells were exposed to UVB or H_2_O_2_, washed twice with PBS, and replenished with a complete culture medium. The cells were then continuously cultured at 37°C and 5% CO_2_ for another 24 h. Finally, the enzyme activities were assessed using SOD, CAT, and GSH‐Px enzyme activity detection kits.

### 2.12 MMP‐1, TIMP‐1 Expression, and Photo Damage Repair Assay

HSF cells were utilized in this experiment to assess their ability to express MMP‐1 and TIMP‐1. Initially, the cells were cultured to a specific density and then incubated with MB (133.3 μg/mL), LB (235 μg/mL), BRM (20 μg/mL), bio‐vesicles (113.3 μg/mL), retinol (20 μg/mL), and trifluoroacetyl tripeptide‐2 (20 μg/mL). After postprocessing, the expression levels of MMP‐1 and TIMP‐1 were tested. A photodamage repair assay was then conducted on the cells following UV irradiation. The dosages of the various active agents were consistent with those previously mentioned. Once the cell density reached the required level, the cells were exposed to UV light of appropriate intensity to create a photodamage model. The test actives were then diluted in serum‐free medium and added to the culture dish. The cells were cultured at 37°C with 5% CO_2_ for 48 h. Subsequently, the cells were fixed with paraformaldehyde and stained with hematoxylin, Ponceau‐Fuchsin, and aniline blue. The fiber density, orientation, and thickness were observed under a microscope, and the fiber area per unit cell was calculated. The expression levels of MMP‐1, TIMP‐1, and Col‐I were also tested by Elisa kit according to the standard process.

### 2.13 Scratch Repair Assay

The HSF cells were used in the scratch repair experiment, which was divided into six groups: Control, UVB, MB (133.3 μg/mL), LB (235 μg/mL), BRM (20 μg/mL), and bio‐vesicles (113.3 μg/mL). The HSF cells were seeded into a six‐well plate. Once the cell density reached 80%, a straight line was scratched vertically on the culture plate using a 200‐μL pipette. The cells were then incubated with cell culture medium, MB, LB, BRM, and bio‐vesicles for 24 h. Subsequently, the cells were exposed to UVB, washed twice with PBS, and supplemented with a complete culture medium. Afterward, the cells were continuously cultured at 37°C and 5% CO_2_. Digital photographs of scratches were taken at 0, 6, 12, and 24 h.

### 2.14 Clinical Research

A prospective, randomized, single‐blind, controlled trial was conducted on 70 Chinese women aged between 25 and 65 with visible facial wrinkles (Table 1). Subject had a clinical score of crow's feet ≥ 2, under‐eye wrinkles ≥ 2, nasolabial folds ≥ 1, and forehead wrinkles ≥ 2, assessed at the Shanghai Institute of Nutrition and Health. Dermatologists evaluated all participants for eligibility, provided comprehensive information regarding the study's benefits and risks, and obtained written consent. This study received approval from the Shanghai Ethics Committee (Approval number: ER‐SINH‐262441) and adhered to the ethical principles of the Declaration of Helsinki.

**TABLE 1:** Age and skin type distribution of subjects in different groups.

Consumer profile		Treatment (*n* = 30)	Placebo (*n* = 32)
Average age (y)	Mean ± SD	44.20 ± 5.99	43.72 ± 6.04
Skin type (%)	Dry	0.00%	0.00%
	Normal	0.00%	12.50%
	Oily	6.67%	0.00%
	Combined skin	93.33%	87.50%

Exclusion criteria included pregnancy, breastfeeding, plans for pregnancy, or a history of allergies to cosmetics, severe allergies, systemic diseases, or significant skin conditions. Sixty‐two participants completed all five visits, while eight dropped out due to distance constraints. The tested product was a facial cream containing 2% MB actives (the detailed composition of the cream is seen in Table S1). The placebo facial cream had an identical base formulation but lacked MB actives. Participants were randomly assigned, with half receiving the active cream and the other half receiving the placebo. Subjects were instructed to apply the cream twice daily and to use sunscreen during the day for 4 weeks, with assessments conducted at baseline and at Week 4. Prior to each evaluation, subjects cleansed their faces, dried them with a lint‐free paper towel, and acclimated in a standardized environment (20°C–22°C, 40%–60% humidity) for 30 min. Subsequent evaluations were performed using specific instruments. Skin elasticity, measured by the gross elasticity parameter R2 (Ua/Uf), was assessed using the Cutometer MPA580 from Courage+Khazaka Electronic GMBH. Facial wrinkles in four areas (nasolabial folds, forehead wrinkles, under‐eye wrinkles, and crow's feet) were examined using topographic skin measurement devices (Antera 3D CS: Miravex Ltd., Ireland and Meitu Eve V).

### 2.15 Statistical Analysis

Continuous variables are presented as means ± standard deviations (SD) from at least three independent replicates. For two‐group comparisons, statistical differences were assessed using a two‐sided Student's *t*‐test. A *p*‐value of < 0.05 (*p < 0.05, **p < 0.01, ***p < 0.001) was considered statistically significant, while *p* > 0.05 was deemed not significant (ns). All statistical analyses were conducted using GraphPad Prism 8 Software.

## 3 Results

### 3.1 Physicochemical Characterization of MB

**FIGURE 1:**
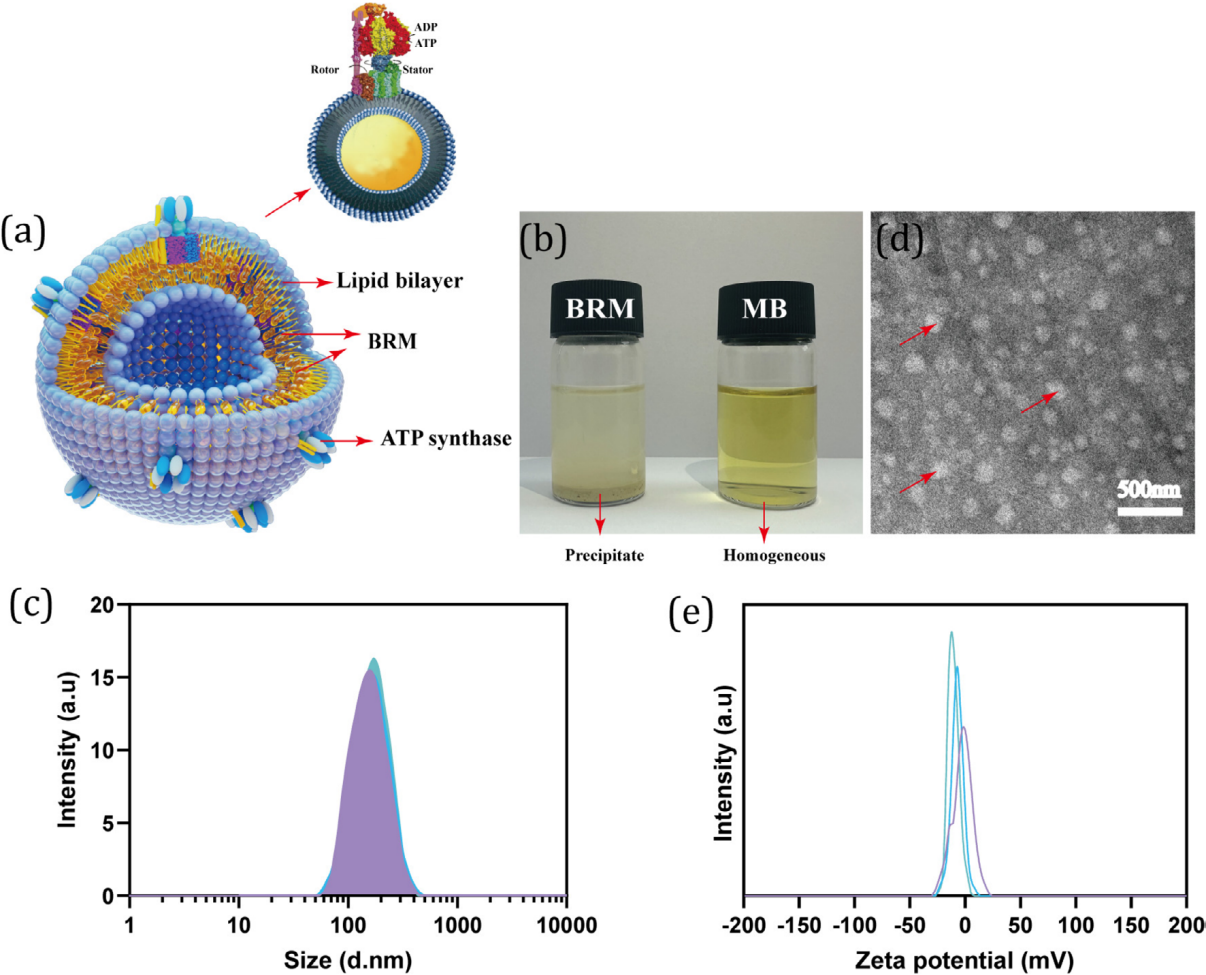
Physical and chemical data of motor baicalin (MB). (a) The structure diagram of MB shows ATP synthase molecules on the surface of bio‐vesicles. The yellow particles dispersed in the hydrophobic phospholipid bilayer present BRM. (b) The solubility of BRM and MB in water. (c) The particle size distributions of three batches of MB. (d) TEM image of MB, where the white circular hollows depict MB vesicles. (e) Zeta potential properties of three batches of MB.

The structure of MB is illustrated in Figure 1a. The hydrophobic baicalin was encapsulated in bio‐vesicles forming a “core‐shell” structure to enhance its water solubility (Figure 1b). Some molecules protrude on the surface of these bio‐vesicles, which are ATP synthase. ATP synthase, a rotating molecular motor found in bacteria, chloroplasts, and mitochondria, drives MB to actively transfer and integrate into specific skin cells, such as HaCAT or fibroblasts. Additionally, the particle size of MB (approximately 145 nm) was confirmed by photon correlation spectroscopy, showing a low polydispersity (PDI) index of 0.18, indicative of a homogeneous system (Figure 1c and Table 2). The particle size and PDI index were the same as the previously reported baicalin nanocarrier system as well as some other drug delivery systems in cosmetics [19, 20, 21, 22]. The results are consistent with the TEM images (Figure 1d). Furthermore, the zeta potential values for multiple batches of MB were tested at approximately −3.41 mV, suggesting good safety and long‐term stability (Figure 1e and Table 2).

**TABLE 2:** Characteristics of baicalin delivery system of MB.

	Parameters	Batch 1	Batch 2	Batch 3	Average value
Particle size	Z‐Average, d.nm	147.6	145.6	144.6	145.9
	PDI	0.21	0.17	0.17	0.18
Zeta potential	Zeta potential, mV	−2.71	−2.97	−4.54	−3.41
Loading capacity	Percentage, %	14.70	15.20	14.80	14.90

The baicalin loading capacity was further investigated using HPLC. Results showed that the average loading rate for three batches is approximately 14.90 wt% (Table 2), which exceeds the LB values (approximately 8.5 wt%). Moreover, the structural stability of MB is crucial for practical application. Consequently, we also tested its long‐term stability over 90 days. Results demonstrated no significant changes in MB's pH values, baicalin loading capacity, particle size, or zeta potential values at different temperatures (Figure S3), further revealing the good long‐term stability of MB.

### 3.2 In Vitro Release Assessment

The in vitro drug release measurement is a crucial method for evaluating the efficacy of bio‐vesicle delivery technology and estimating its in vivo performance qualitatively. In this study, we assessed the release kinetics of MB using the Franz diffusion cells method. For comparison, BRM and LB were also evaluated. As illustrated in Figure S4, a burst release effect was observed within the first 4 h of BRM application, with approximately 60% of baicalin being released. This rapid release could lead to skin sensitivity due to local hyper‐concentration. Indeed, high concentrations of BRM exhibited significant cytotoxicity toward both HaCaT and HSF cell lines (Figure S5). In contrast, both MB and LB demonstrated slow‐release behavior during this initial period, approximately 39% and 29% of baicalin were released from MB and LB, respectively, within the first 4 h. These findings suggest that both MB and LB samples conform to a first‐order release model, extending the release time of the active substance may effectively mitigate potential cytotoxicity and skin irritation risks. This phenomenon can be attributed to the fact that when used as potent antioxidants or whitening agents in cosmetics, polyhydroxyl structures or acid anhydride forms of baicalin may compromise the skin's barrier function. When extending the observation period to 60 h, it was found that the release ratio for MB reached 88%, whereas LB only achieved 64%. This indicates that MB exhibits superior release efficiency compared to LB, which is also very crucial for their realization of antiaging efficacy.

### 3.3 Transdermal Penetration and Endocytosis Evaluation

**FIGURE 2:**
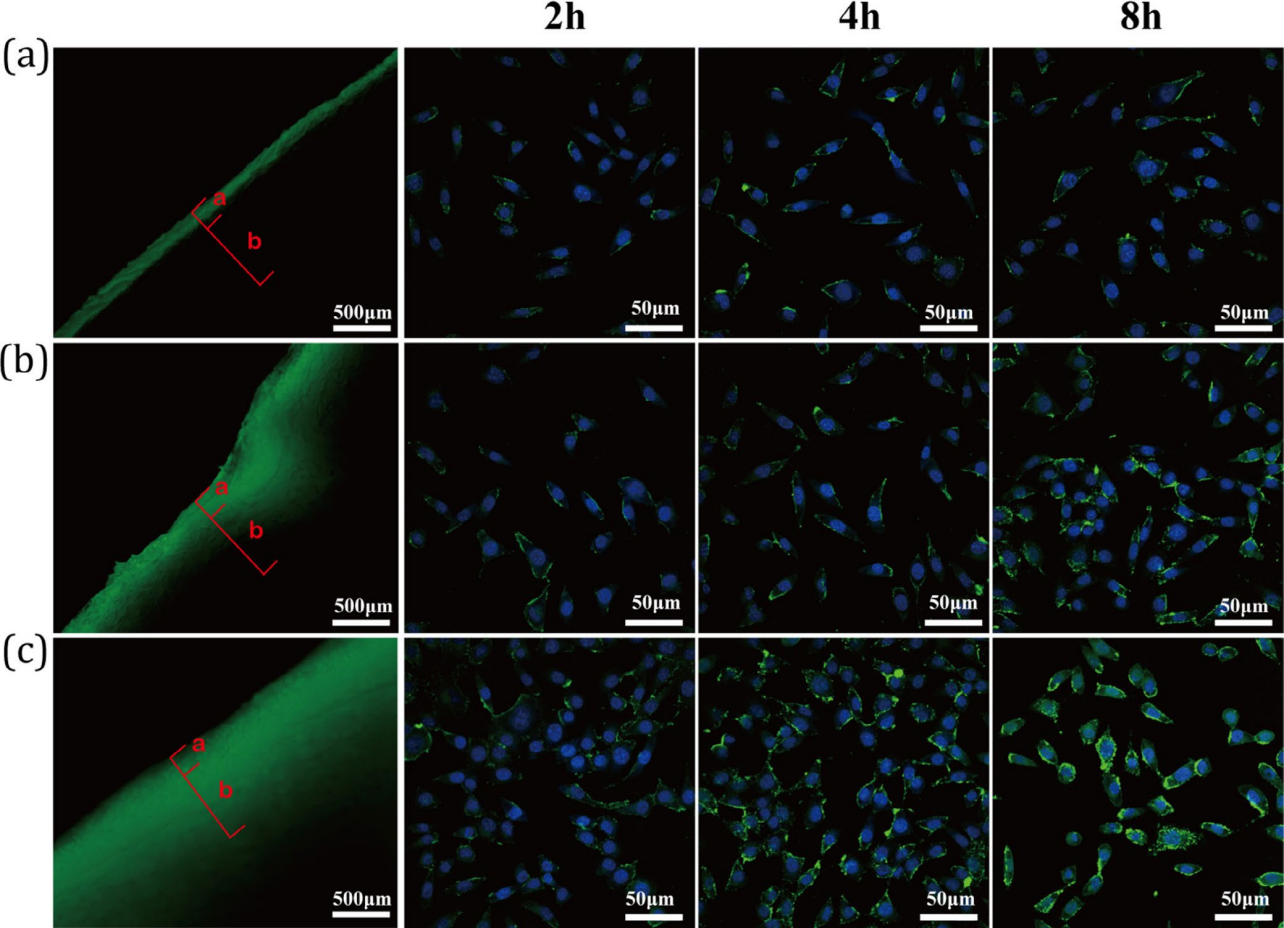
Transdermal penetration and cellular uptake properties of (a) BRM, (b) LB, and (c) MB. In the figure, the red labels “a” and “b” denote the epidermis and dermis, respectively.

To investigate the potential efficacy of MB in cosmetics, an in vitro test was conducted to examine transdermal penetration and endocytosis performance. For comparison, BRM and LB were also evaluated. As illustrated in Figures 2 and S7, BRM demonstrated notably poor transdermal permeability, with a penetration depth of only 264 ± 2 μm, remaining primarily on the skin's surface as evidenced by fluorescence imaging. In contrast, following modification with lipid and bio‐vesicle technology, LB and MB demonstrated enhanced skin penetration capabilities of 555 μm and 1347 μm, respectively, indicating a significant improvement in penetration. Unlike traditional liposome technology and static vesicle encapsulation methods, the primary advantage of ATP molecular motors lies in their ability to undergo rotational disturbance upon receiving biological signals. When the ATP molecular motor bio‐vesicles contact cell membrane, this rotational disturbance further facilitates the fusion between biofilms and enhances the delivery efficiency of active molecules. Consequently, utilizing ATP molecular motor bio‐vesicles to encapsulate baicalin markedly improves its skin penetration and cellular uptake ability, thus significantly amplifying its antiaging effects.

The ability of active ingredients to penetrate the skin is crucial, but their effectiveness also largely depends on their capacity to bind to cells. Therefore, we conducted an endocytosis experiment using L929 fibroblasts. Similar to the transdermal results, the BRM sample was not captured by the cells, owing to its poor permeability, as evidenced by the almost complete absence of fluorescence in the cytoplasmic, even after 8 h of coincubation. In contrast, the cells displayed a relatively strong uptake of LB and MB actives, especially the MB actives. Compared with the LB actives, a substantial amount of baicalin was found in the cytoplasm of the MB. The remarkable uptake of MB is possibly derived from the ATP synthase units on the bio‐vesicles, as ATP synthase can act as a molecular motor, driving the bio‐vesicles that carry baicalin to integrate into specific skin cells.

### 3.4 Effect of MB Against Cell Damage Caused by Oxidative Stress

**FIGURE 3:**
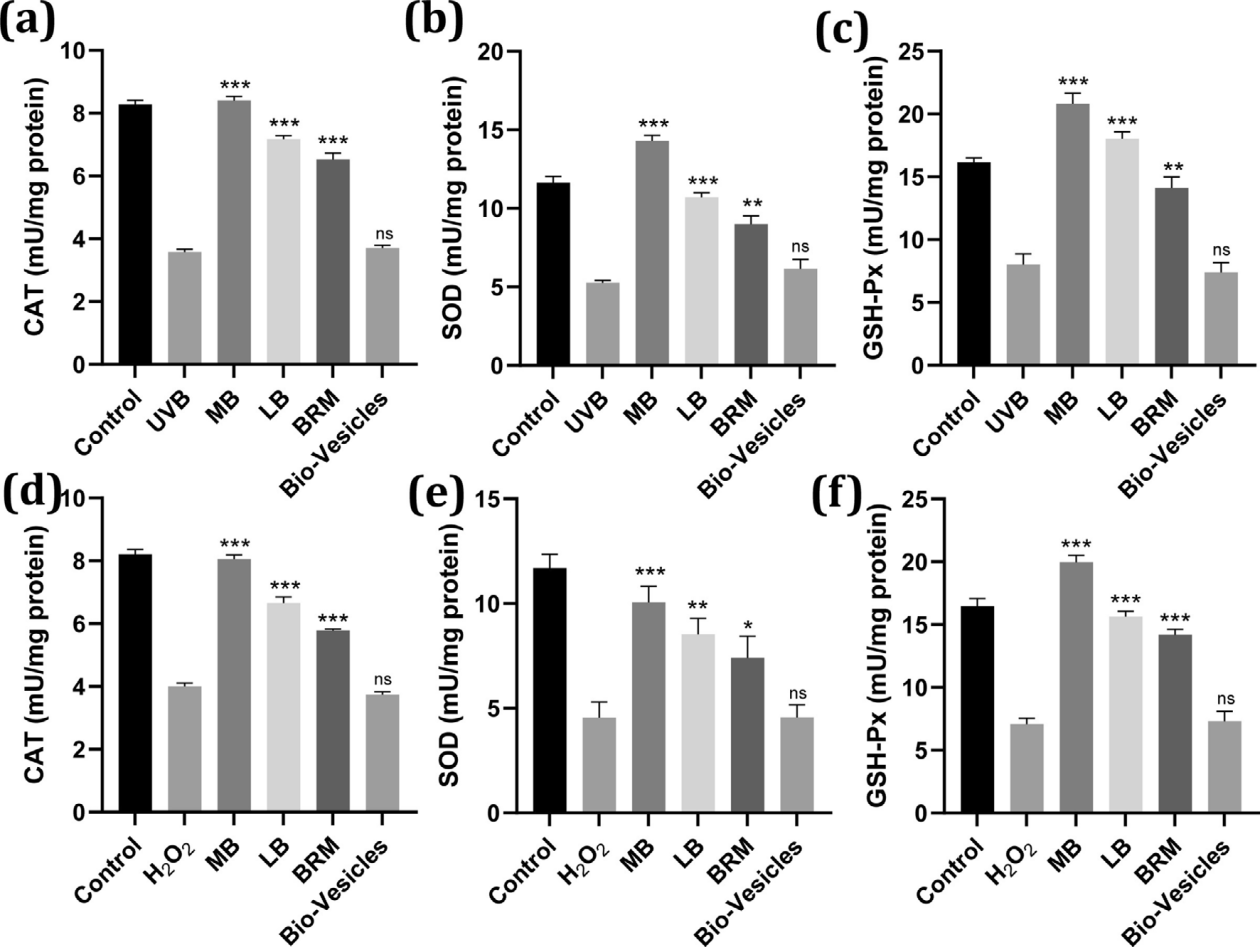
Antioxidant capabilities of various baicalin‐related actives and bio‐vesicles. Panels (a, d) depict CAT, (b, e) show SOD, and (c, f) represent GSH‐Px gene expression in HSF cells. Data are reported as mean values ± SD. The symbol * indicates values that are significantly different from those of the UVB or H_2_O_2_ groups (*p < 0.05, **p < 0.01, ***p < 0.001).

The CCK‐8 assay of bio‐vesicles and baicalin‐related actives in different concentrations confirmed that bio‐vesicles and baicalin‐related actives exhibited excellent bio‐compatibility (Figure S5). The cell viability of MB, LB, BRM, and bio‐vesicles exposed to UVB and H_2_O_2_ is shown in Figure S6. The results exhibited that the antioxidant efficacy of MB was superior to LB and BRM, which can be attributed to the enhanced delivery efficiency of BRM by ATP synthase molecular motor technology. Then, we further investigated the relative activity changes of three key intracellular enzymes: SOD, CAT, and GSH‐Px, which are strongly correlated with cellular endogenous antioxidant capacity. As shown in Figure 3, the activities of SOD, CAT, and GSH‐Px were significantly reduced to less than 50% compared to the control cells (*p* < 0.05) when exposed to UVB or H_2_O_2_, indicating severe oxidative damage. Subsequently, treatment with baicalin‐related actives markedly improved the expression of these enzymes, particularly in the MB group. The activities of SOD, CAT, and GSH‐Px in the MB group increased to 101.5%, 122.9%, and 128.9% in the UVB model and to 98.2%, 86.2%, and 121.3% in the H_2_O_2_ model, respectively (*p* < 0.05), compared to the blank control group, demonstrating the excellent antioxidant potential of MB actives. This result is consistent with the immunofluorescence test findings for UVB (Figure S8) and H_2_O_2_ (Figure S9) as ROS stimuli.

### 3.5 Effect of MB on Antiaging Evaluation

**FIGURE 4:**
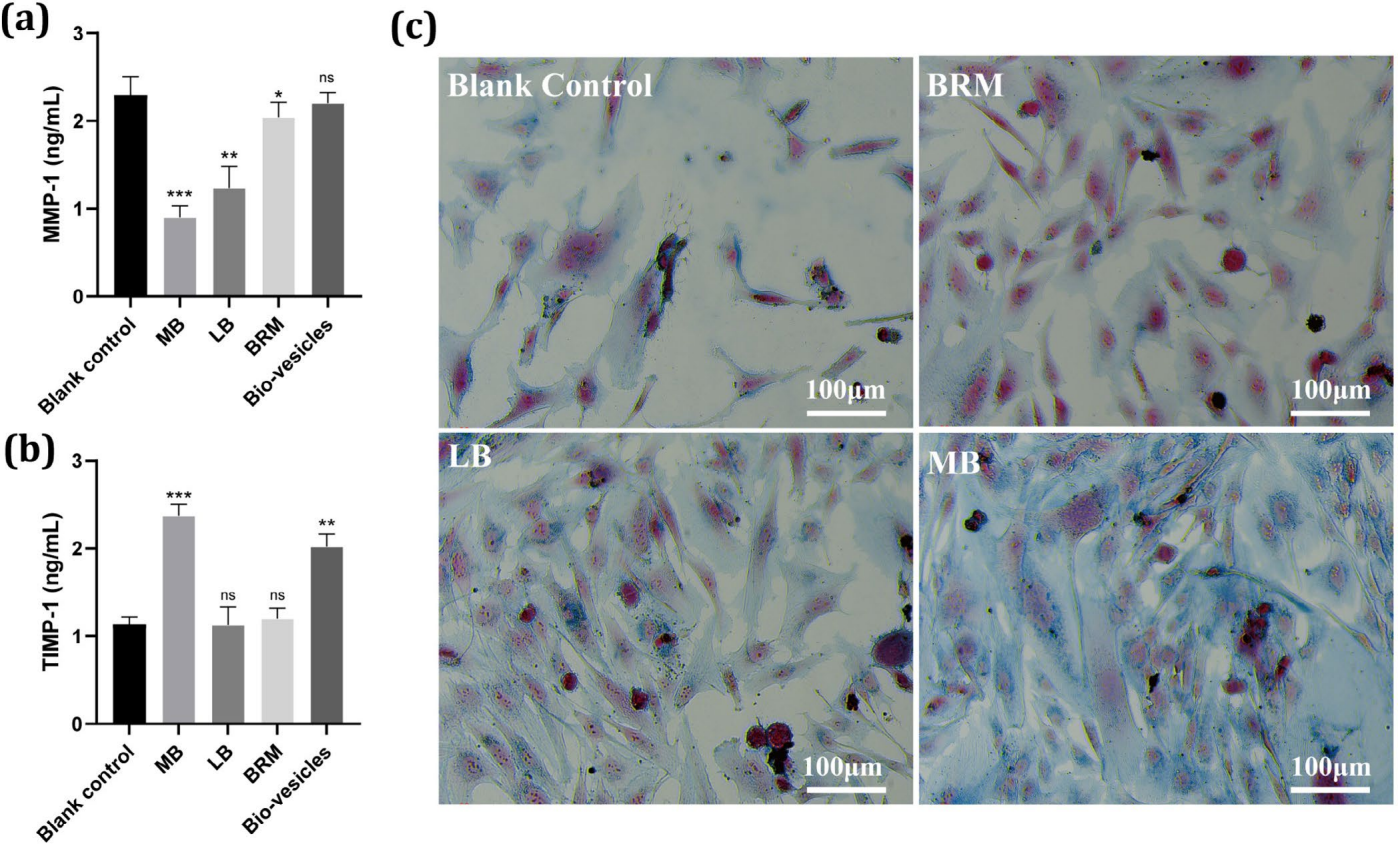
Antiaging properties of various baicalin‐related actives and bio‐vesicles. (a, b) MMP‐1 and TIMP‐1 gene expression in HSF. Data are presented as mean values ± SD. An asterisk (*) indicates values that are significantly different from the blank control (*p < 0.05, **p < 0.01, ***p < 0.001). (c) Cell fibrous staining images of BRM, LB, and MB actives. The blue regions represent the fibrous structures of HSF, while the red regions denote the fibroblasts themselves.

Matrix metalloproteinase‐1 (MMP‐1) and tissue inhibitor of metalloproteinase‐1 (TIMP‐1) are a pair of enzymes in our skin that regulate the expression and degradation of collagen, playing a crucial role in skin aging. To explore the antiaging properties of baicalin, we examined the activity changes of MMP‐1 and TIMP‐1 in baicalin‐related actives. As shown in Figure 4a,b, when cells were incubated with test samples, all baicalin‐related groups, including BRM, MB, and LB, demonstrated a significant inhibitory effect on the MMP‐1 gene compared to the blank control group (*p* < 0.05), except for the bio‐vesicles group. Among these, the MB active exhibited the most substantial inhibition, indicating excellent collagen protection potential. Similarly, MB actives also showed the most pronounced promotion of TIMP‐1 expression, further confirming their superior collagen‐protective and antiaging capabilities. Furthermore, to elucidate the exceptional collagen‐protective performance of MB more clearly, commonly used antiaging ingredients such as retinol and trifluoroacetyl tripeptide‐2 were evaluated for comparison purposes. The results disclosed that MB exhibits comparable or even superior inhibition of MMP‐1 while promoting TIMP‐1 activity relative to both retinol and trifluoroacetyl tripeptide‐2 (Figure S10). In addition, the protein levels of MMP‐1 and TIMP‐1 treated with BRM, MB, LB, and bio‐vesicles were confirmed by ELISA (Figure S11a,b). This suggests that MB‐based ATP molecular motors enhance the delivery of baicalin effectively, bolstering collagen protection and antiaging properties.

### 3.6 Effect of MB on Repairing of UV‐Damaged Fibroblasts

Fibroblasts are a pivotal component of skin tissue, and the density, thickness, and orientation of fibrous structures within them predominantly reflect the cellular state and degree of aging. UV radiation can thin collagen fibers within cells, thereby accelerating cellular aging. In this study, we further investigated the reparative and promoting effects of baicalin on fibroblasts post‐UV irradiation. Human skin fibroblasts were used in the experiment; for details, see the experimental section. After UV exposure, the fibrous structures in the blank control group became thinner and exhibited abnormal orientation, indicating accelerated cellular aging due to UV irradiation (Figure 4c). Upon incubating UV‐damaged cells with baicalin‐related actives, a significant improvement in cell vitality was observed. The fibrous structures within the cells appeared thicker compared to the blank control. This suggests that all three baicalin‐related actives have a reparative effect on photoaged human skin fibroblasts. Among them, the MB actives showed the most significant enhancement, evidenced by a large amount of blue collagen fibers generated around the cells. This may be attributed to the strong uptake effect of fibroblasts on MB actives. In addition, the expression protein level of Col‐I also confirmed that all three baicalin‐related actives have a reparative effect on photoaged HSF by increasing Col‐I (Figure S11c).

### 3.7 Effect of MB on Wound Healing

**FIGURE 5:**
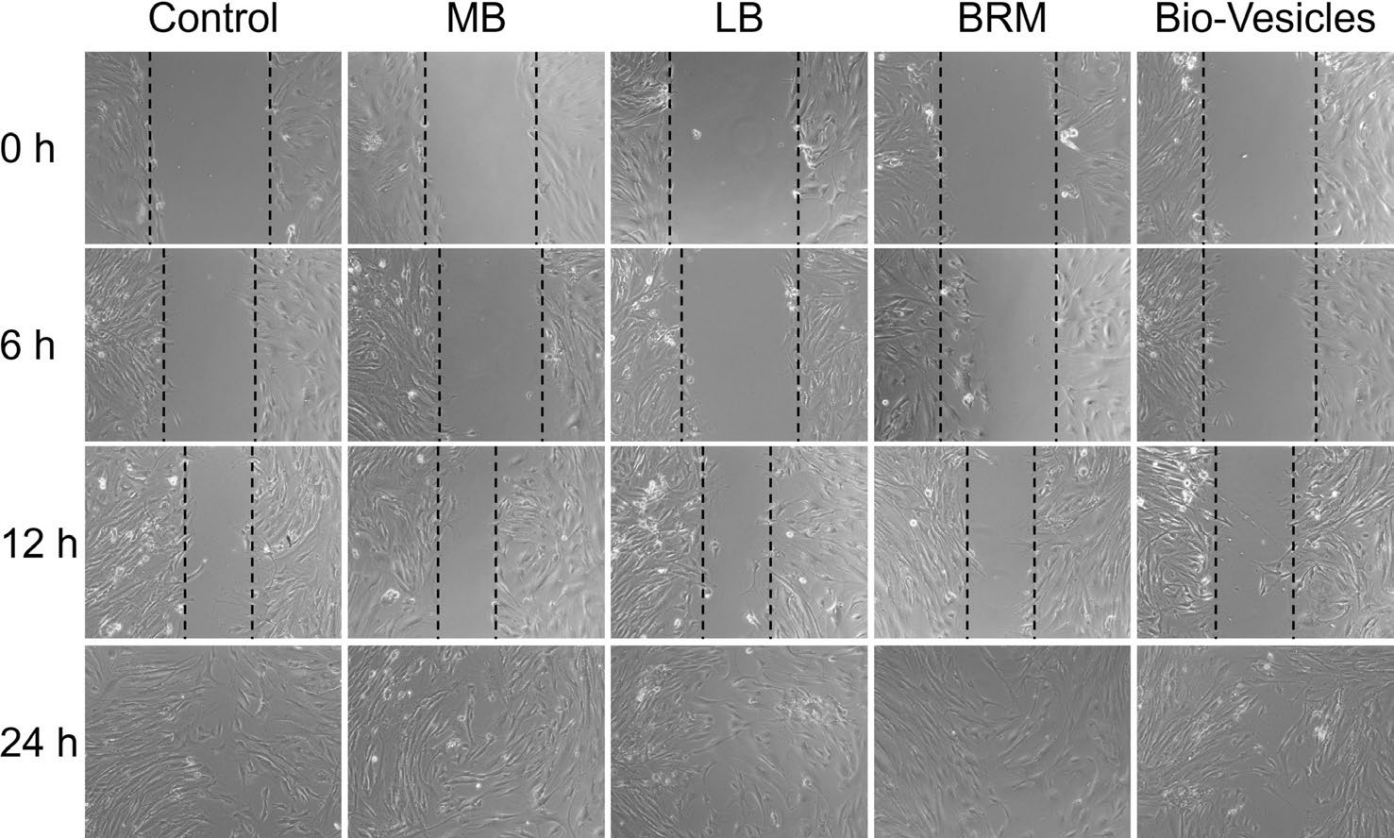
The Effect of MB on wound closure. Images of scratch assays performed on HSF monolayers were treated with control, UVB, MB, LB, BRM, and bio‐vesicles groups for 0, 12, 24, and 36 h. Scale bar: 100 μm. The results show that MB enhances the rate of wound closure.

Enhanced fibroblast migration is crucial for effective skin healing and regeneration, particularly in aged or photodamaged skin. By facilitating faster wound closure, enhancing collagen production, and restoring skin structure and elasticity, fibroblast migration plays a significant role in overall skin health. The application of therapies designed to enhance fibroblast function presents a promising strategy for improving the appearance and quality of aging skin. Therefore, the scratch assay was performed on HSF cells to evaluate the effects of three baicalin‐related actives on cell spread in the wound area. As shown in Figure 5, the migration of HSF cells was calculated to be 53.8%, 79.8%, 59.2%, 52.5%, and 54.7% for control, MB, LB, BRM, and bio‐vesicles group, respectively. Compared to BRM and LB actives, the MB actives showed a superior ability to promote HSF cells move to scratch areas, indicating that MB could increase the migration ability of HSF cells. Therefore, baicalin can significantly enhance cell vitality, combined with its ability to repair fibers, suggesting its great potential in antiaging field.

### 3.8 Clinical Research

**FIGURE 6:**
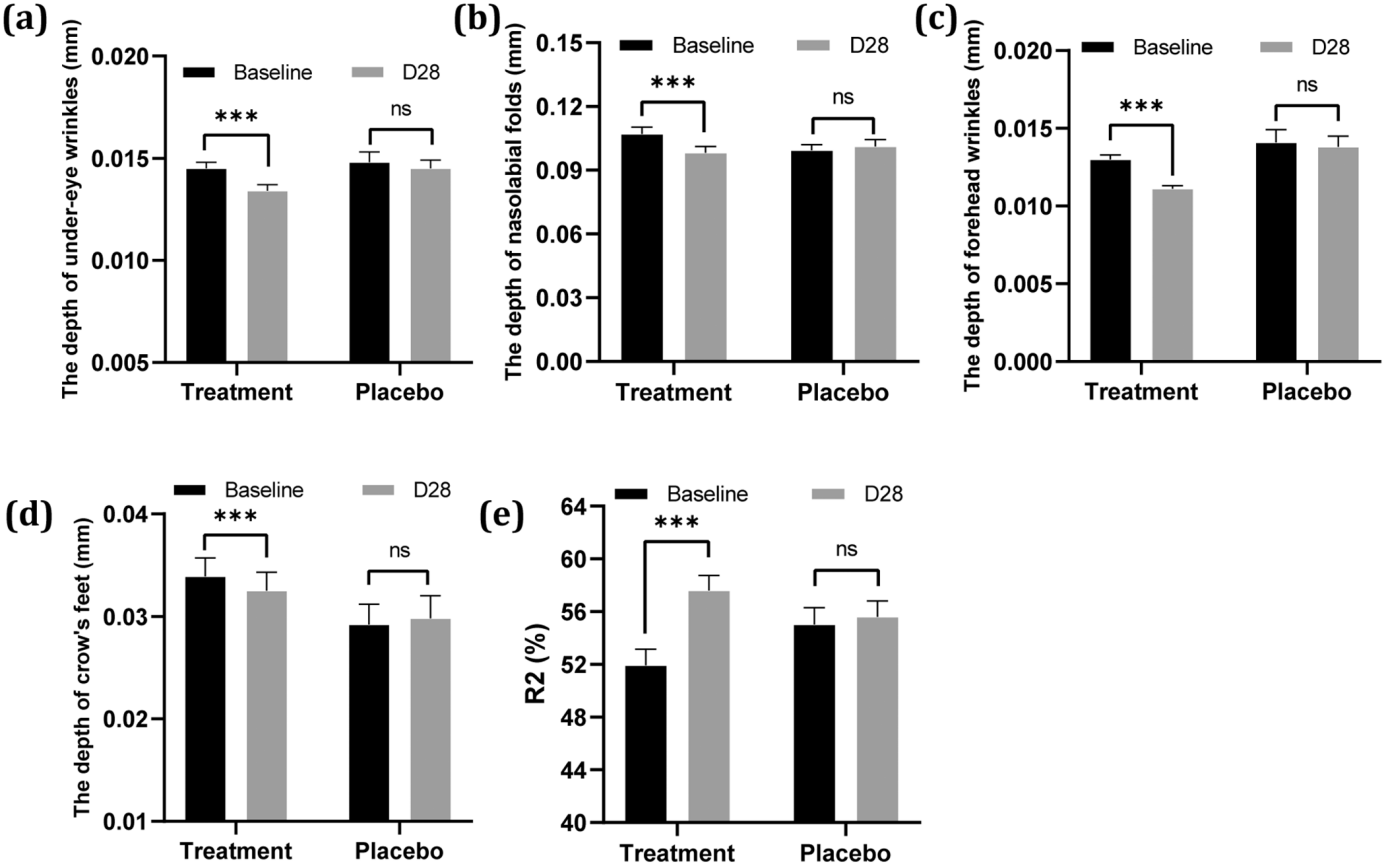
Efficacy of the facial cream in reducing wrinkles and improving skin elasticity. (a) Reduction in under‐eye wrinkles, (b) Reduction in nasolabial wrinkles, (c) Reduction in forehead wrinkles, (d) Reduction in crow's feet, (e) Improvement in skin elasticity) (***p ≤ 0.001 vs. baseline).

**FIGURE 7:**
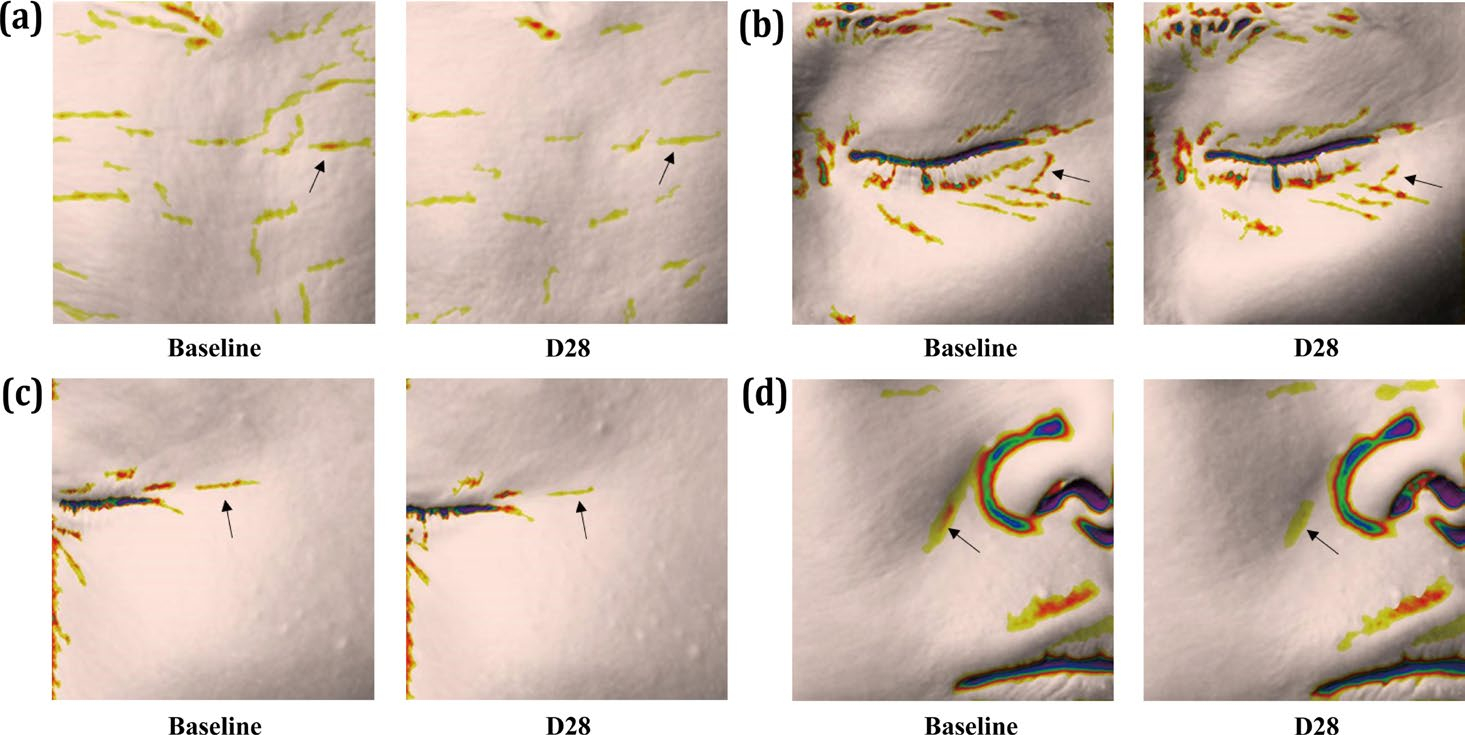
The examples of in vivo antiaging efficacy of the eye cream. (a) Forehead wrinkles reduction, (b) Under‐eye wrinkle reduction, (c) Crow's feet reduction, (d) Nasolabial folds reduction).

**FIGURE 8:**
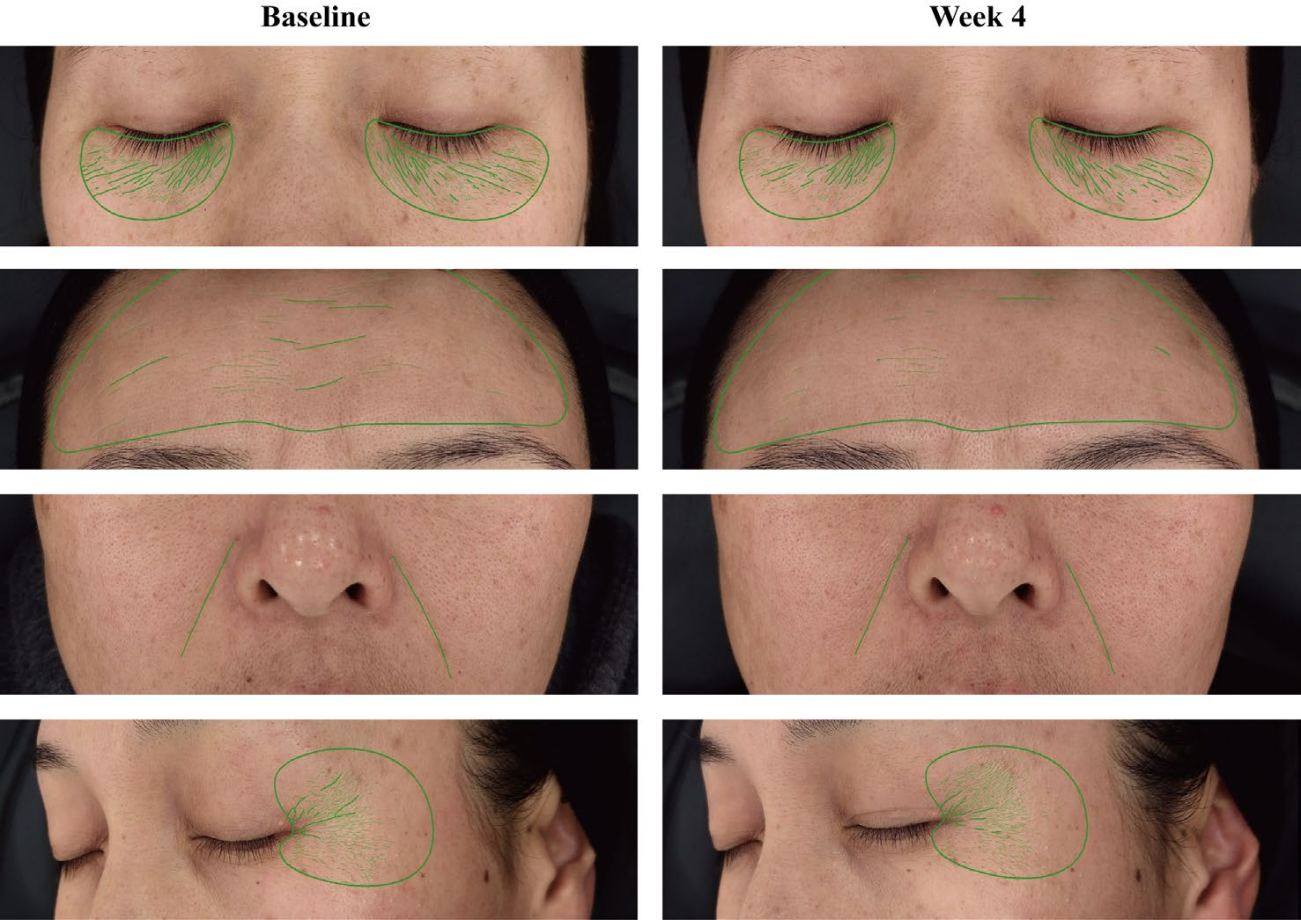
Analysis of nasolabial wrinkles, under‐eye wrinkles, forehead wrinkles, and crow's feet in participants following supplementation treatment. Representative Meitu images taken at baseline and after 4 weeks of supplementation.

Given the excellent antiaging potential demonstrated by MB actives in vitro, we conducted clinical research to further validate these findings. The in vivo instrumental evaluation results are summarized in Figure 6. Compared to the baseline, the target group with 2% MB additives showed significant benefits in reducing wrinkles and improving elasticity. According to Antera 3D data, there was a notable reduction of 8.30%, 7.06%, 7.43%, and 4.27% in the depth of nasolabial folds, forehead wrinkles, under‐eye wrinkles, and crow's feet, respectively, on Day 28. Additionally, the length of nasolabial folds, marionette lines, and under‐eye wrinkles decreased by 3.46%, 9.59%, and 32.31%, respectively. These effects were significantly different from the placebo at both intervals. Furthermore, an increase of 10.92% in the *R* ^2^ value, indicating improved skin elasticity, was observed in the 2% MB additives group on Day 28 compared to the placebo. Figures 7 and 8 also provide illustrative examples highlighting the antiwrinkle efficacy of the MB actives in the facial cream.

## 4 Discussion

In this study, we developed a novel baicalin delivery system, named Motor Baicalin (MB), to address the challenges of insolubility, instability, and low skin permeability associated with baicalin. The MB system employs bio‐vesicles derived from yeast and bacillus cell membranes to encapsulate baicalin, forming a core‐shell structure that enhances its water solubility and stability. Our findings indicate that the MB system markedly enhances the bioavailability, antioxidant capacity, and antiaging properties of baicalin. This system outperforms both BRM and commonly used LB formulations while demonstrating comparable efficacy to other previously established baicalin delivery systems [19, 20, 21, 22, 23, 24].

The physicochemical characterization of MB revealed a particle size of approximately 145 nm with a low polydispersity index, indicating a homogeneous system. The zeta potential values suggested good safety and long‐term stability, further confirmed by the absence of significant changes in pH, baicalin loading capacity, particle size, and zeta potential over 90 days. These properties are crucial for practical application, ensuring that MB can maintain its effectiveness over extended periods. Moreover, the in vitro release assessment demonstrated that MB exhibited a slower and more controlled release of baicalin compared to BRM and LB, resulting in higher cumulative release over 60 h. This slow‐release behavior reduces the risk of local hyperconcentration and associated skin sensitivity, providing a safer and longer lasting efficacy. Additionally, transdermal penetration studies showed that MB significantly enhanced the penetration of baicalin into the deeper layers of the dermis compared to BRM and LB. This improved penetration is likely due to the ATP synthase units on the bio‐vesicles, which facilitate active transfer and integration into specific skin cells [16, 25].

The antioxidant efficacy of MB was evaluated by measuring the activities of key intracellular enzymes (SOD, CAT, and GSH‐Px) under oxidative stress conditions. The results indicated that MB significantly enhanced the activities of these enzymes, highlighting its strong antioxidant potential [26]. Moreover, MB exhibited superior antiaging properties by inhibiting MMP‐1 expression and promoting TIMP‐1 expression, suggesting a promising collagen protection ability [27]. These findings were corroborated by the improved cell vitality and fibrous structure observed in UV‐damaged fibroblasts treated with MB.

The wound‐healing assay demonstrated that MB facilitated faster wound closure compared to BRM and LB, likely due to its superior cell bioavailability and its ability to stimulate cell proliferation and migration [28]. Clinical research further validated the antiaging potential of MB actives, showing significant reductions in wrinkles and improvements in skin elasticity in participants using a facial cream with 2% MB additives. These findings underscore the potential of MB as a highly effective cosmetic ingredient for antiaging and skin repairing.

## 5 Conclusions

Our study introduces a promising baicalin delivery system that effectively addresses the primary challenges of insolubility, instability, and low skin permeability. The MB system not only enhances the bioavailability and stability of baicalin but also boosts its antioxidant, antiaging, and wound‐healing properties. Clinical findings further validate the efficacy of MB in reducing wrinkles and improving skin elasticity, making it a valuable addition to cosmetic formulations. Future research should explore the long‐term effects of MB in clinical settings and investigate its potential applications in other skin‐related conditions.

## Author Contributions

L.C., F.X., and Y.G. contributed to conceptualization. F.X. and Y.G. developed the methodology. L.C. and F.W. were responsible for the software. Y.G., F.W., and N.L. conducted the formal analysis. L.C. and F.W. took part in the investigation. L.X., M.X., and L.C. were responsible for resources. L.X., M.X., and L.C. were responsible for data curation. L.C. and F.W. contributed to writing – original draft preparation. L.C., F.W., and F.X. were responsible for writing – review and editing. L.C. and N.L. were involved in visualization. L.X., M.X., and L.C. were involved in supervision. All authors have read and agreed to the published version of the manuscript.

## Ethics Statement

The study was conducted in accordance with the Declaration of Helsinki and approved by the Shanghai Ethics Committee (ER‐SINH‐262441).

## Consent

Informed consent was obtained from all subjects involved in the study.

## Conflicts of Interest

The authors declare no conflicts of interest.

## Supporting information


Appendix S1.

## Data Availability

The data that support the findings of this study are available from the corresponding author upon reasonable request.
