# Supplementary material for: Transdermal Delivery of Baicalin Based on Bio‐Vesicles and Its Efficacy in Antiaging of the Skin
Source: J Cosmet Dermatol. 2025 Feb 13;24(2):e70024. doi: 10.1111/jocd.70024 (PMC11824916; doi:10.1111/jocd.70024)
Supplement: Supplementary file 1 — Appendix S1. [file JOCD-24-e70024-s001.docx]

Supplementary material

Transdermal Delivery of Baicalin Based on Bio-Vesicles and Its Efficacy in Anti-Aging of the Skin


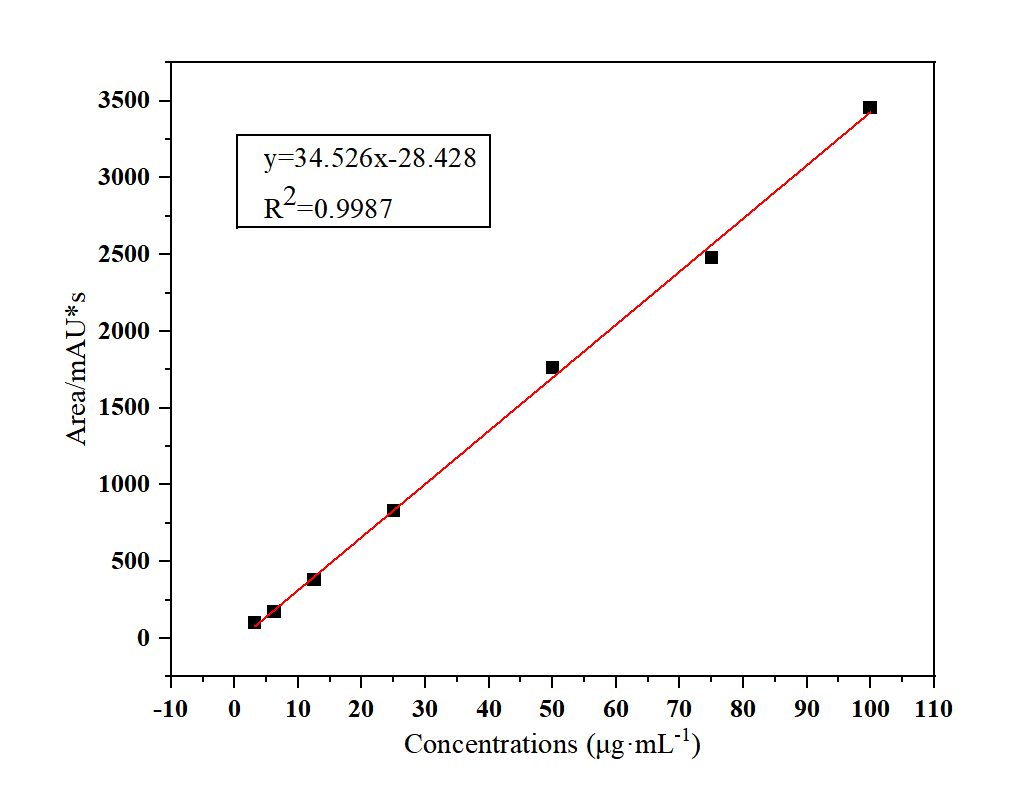


**Figure S1. The standard curve of baicalin with the concentration from 0-100 μg/mL.**

**

**Figure S2. The synthesized routine of FITC labeled baicalin.**


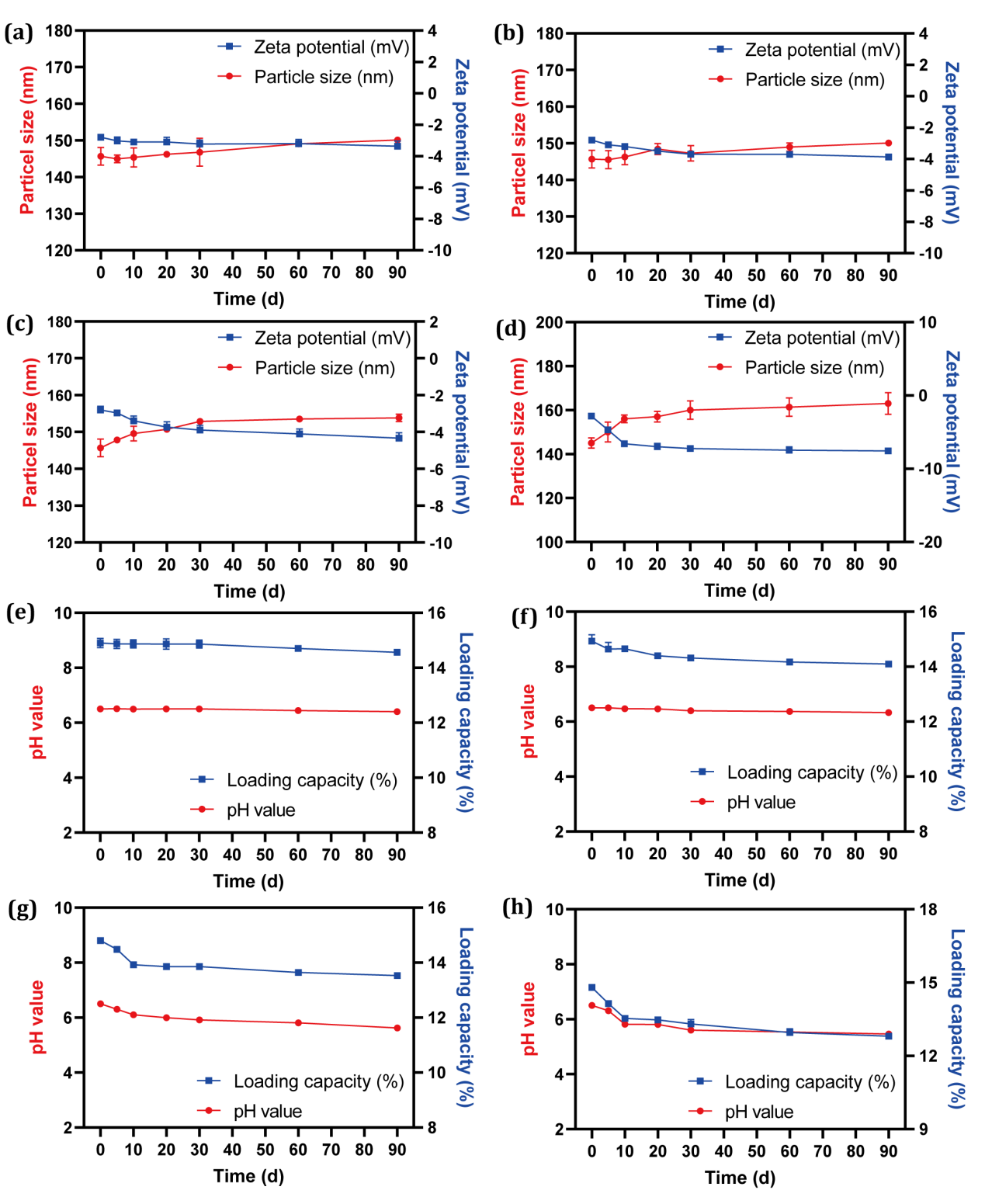


**Figure S3. Stability data of MB. Stability data of zeta potential and particle size within 90 days at different temperature of -20 (a), 4 (b), RT (c) and 40 (d).** Stability data of loading capacity and pH value within 90 days at different temperature of -20 (e), 4 (f), RT (g) and 40 (h).


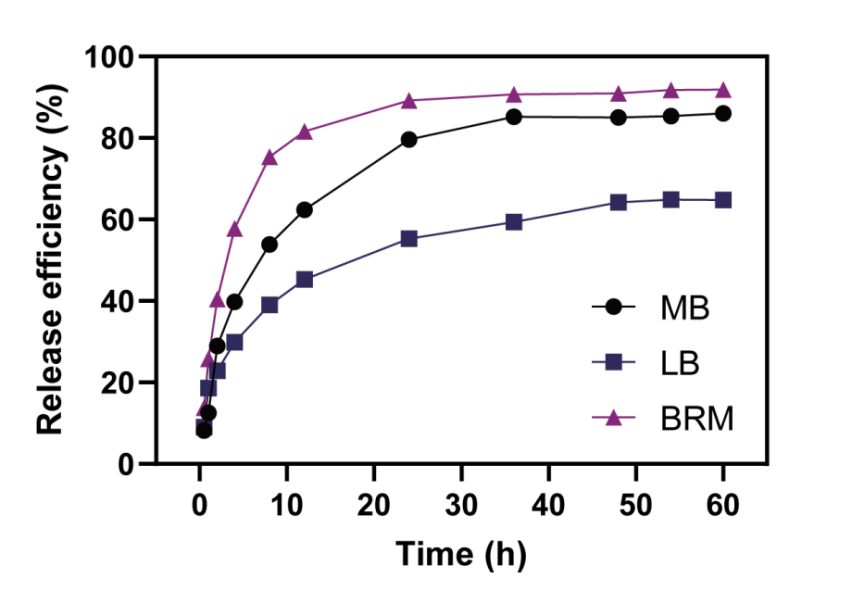


**Figure S4. In vitro release data of baicalin-based actives.**


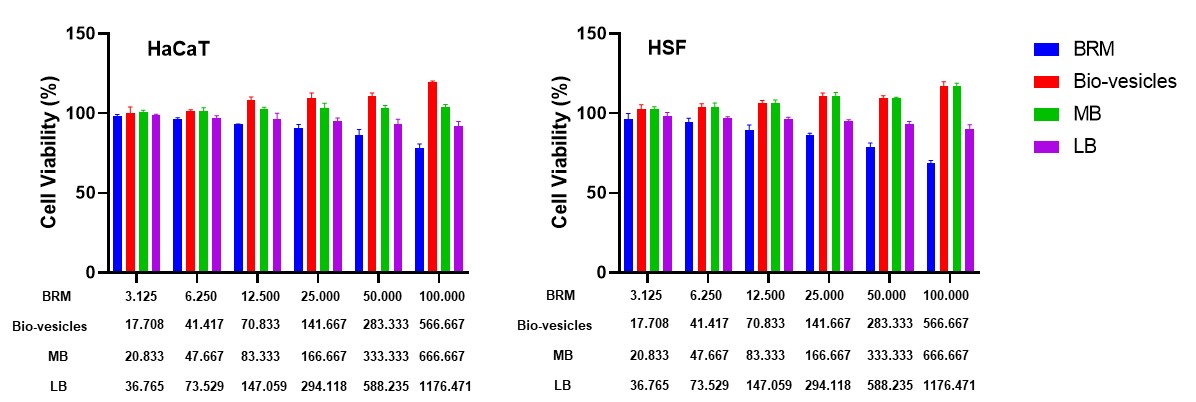


**Figure S5. Cell viability data of baicalin-based actives and bio-vesicles in HaCaT (a) and HSF (b) cell.**


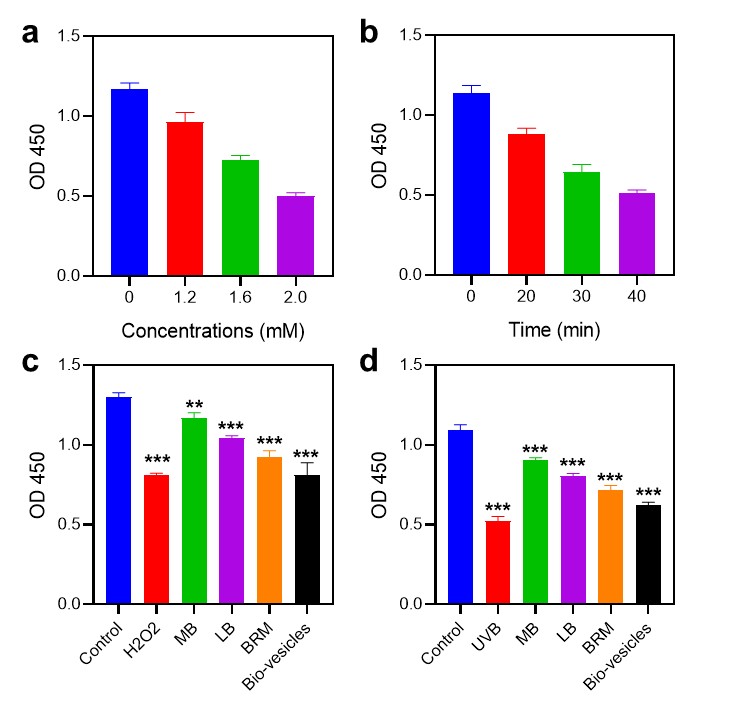


**Figure S6. Cell viability data of H_2_O_2_ for 1.2, 1.6 and 2.0 mM incubated 30 min with cells (a) and exposed with UVB for 20, 30 and 40 min (b); Cell viability data of baicalin-based actives and bio-vesicles in H_2_O_2_ (c) and UVB (d).**

*
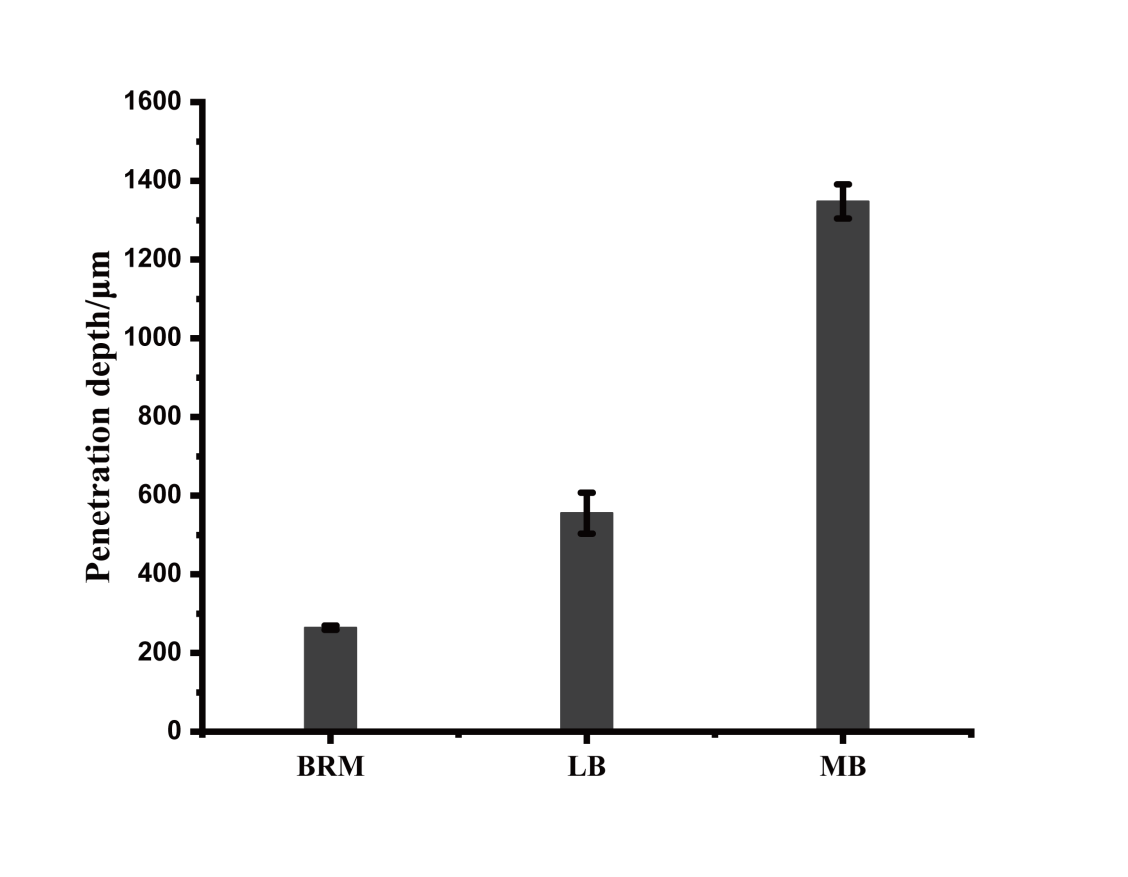
*

**Figure S7. Quantitative data on the penetration depth of BRM, LB and MB into the dermal layers.**


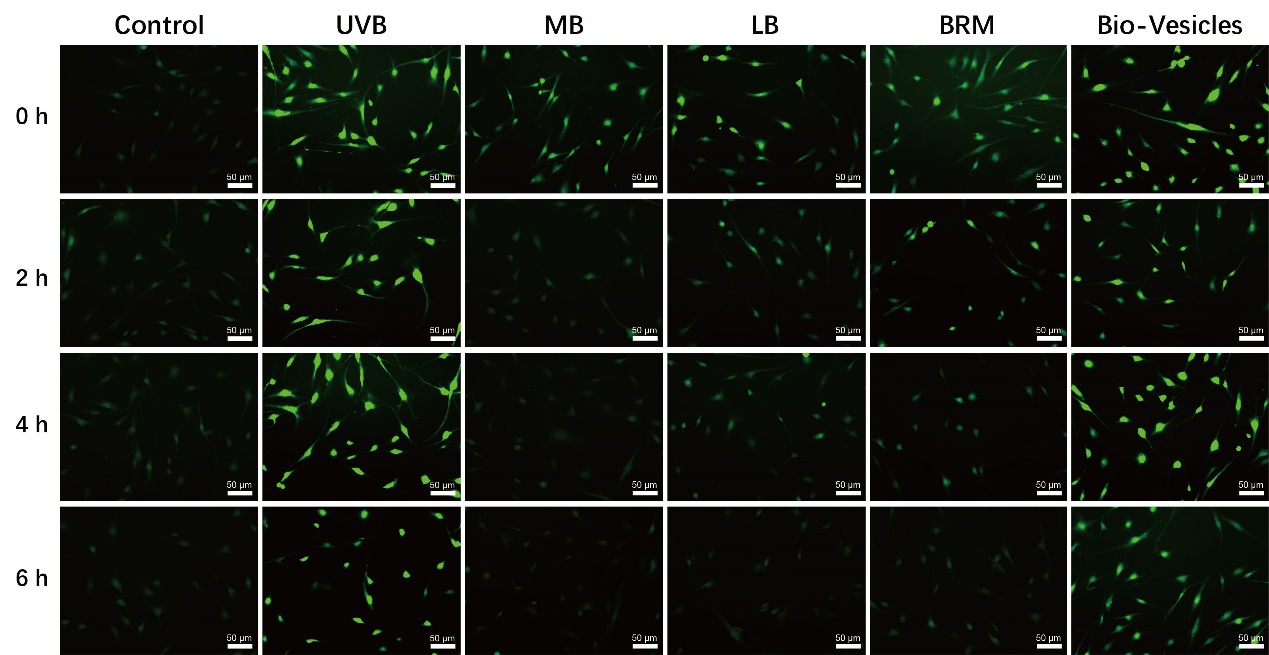


**Figure S8. Representative images of anti-ROS for Control, UVB, MB, LB, BRM and Bio-Vesicles groups by UVB inducing ROS model in HSF cell line.** Scale bar: 50 μm.


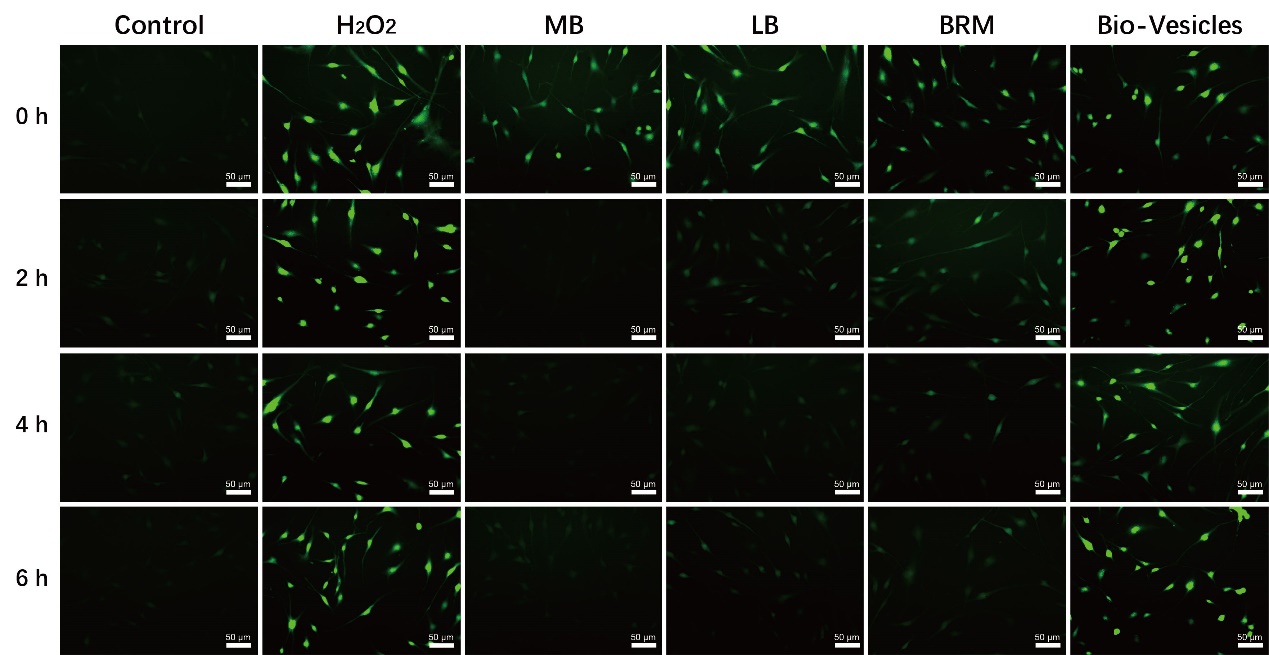


**Figure S9. Representative images of anti-ROS for Control, UVB, MB, LB, BRM and Bio-Vesicles groups by H_2_O_2_ inducing ROS model in HSF cell line.** Scale bar: 50 μm.


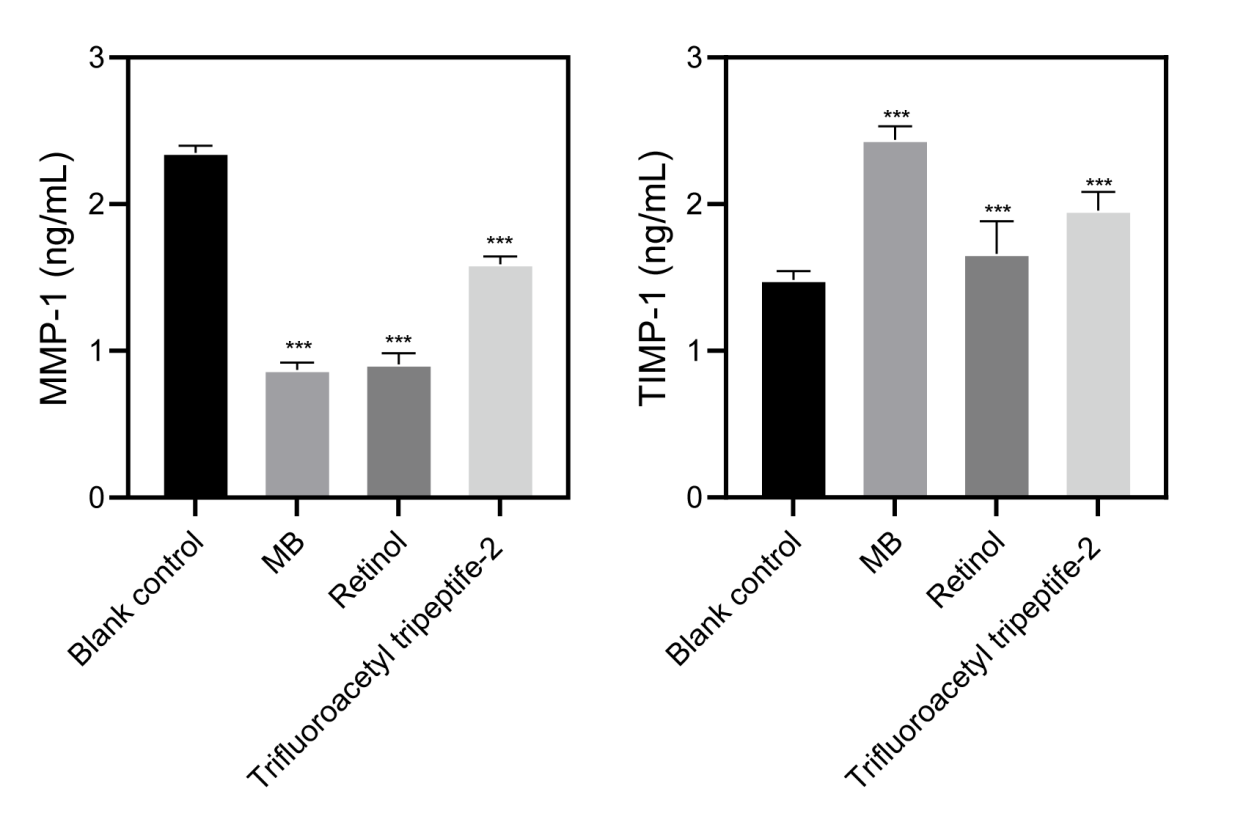


**Figure S10. MMP-1 (a) and TIMP-1 (b) gene expression in HSF. Data are reported as mean values ± standard deviation). Symbol * indicated values which are significantly different from the blank control (* p<0.05, **p<0.01, ***p<0.001).**


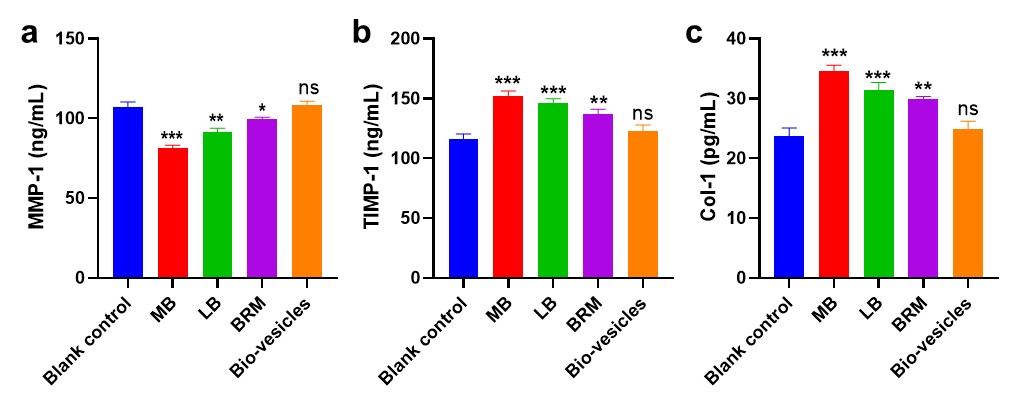


**Figure S11. The expression of MMP-1 (a), TIMP-1 (b) and Col-I in HSF. Data are reported as mean values ± standard deviation). Symbol * indicated values which are significantly different from the blank control (* *p*<0.05, ***p*<0.01, ****p*<0.001).**

**Table S1. The composition of Cream with or without 2% MB**

| **Without 2% MB** | | **With 2% MB** | |
| --- | --- | --- | --- |
| **Ingredient name** | **Content**  **(%)** | **Ingredient name** | **Content**  **(%)** |
| **HYDROXYACETOPHENONE** | **0.5** | **HYDROXYACETOPHENONE** | **0.5** |
| **VINYL DIMETHICONE/METHICONE SILSESQUIOXANE CROSSPOLYMER** | **1** | **VINYL DIMETHICONE/METHICONE SILSESQUIOXANE CROSSPOLYMER** | **1** |
| **CARBOMER** | **0.25** | **CARBOMER** | **0.25** |
| **JOJOBA ESTERS** | **2** | **JOJOBA ESTERS** | **2** |
| **TOCOPHEROL** |  | **TOCOPHEROL** |  |
| **TOCOPHERYL ACETATE** | **0.25** | **TOCOPHERYL ACETATE** | **0.25** |
| **JOJOBA ESTERS** | **2** | **JOJOBA ESTERS** | **2** |
| **TOCOPHEROL** |  | **TOCOPHEROL** |  |
| **GLYCERIN** | **1** | **GLYCERIN** | **1** |
| **WATER** | **66.2** | **WATER** | **64.2** |
| **BUTYLENE GLYCOL** | **5** | **BUTYLENE GLYCOL** | **5** |
| **DIMETHYLMETHOXY CHROMANOL** | **0.05** | **DIMETHYLMETHOXY CHROMANOL** | **0.05** |
| **DISODIUM EDTA** | **0.05** | **DISODIUM EDTA** | **0.05** |
| **DICAPRYLYL CARBONATE** | **5** | **DICAPRYLYL CARBONATE** | **5** |
| **TOCOPHEROL** |  | **TOCOPHEROL** |  |
| **XANTHAN GUM** | **0.05** | **XANTHAN GUM** | **0.05** |
| **BUTYROSPERMUM PARKII (SHEA) BUTTER** | **8** | **BUTYROSPERMUM PARKII (SHEA) BUTTER** | **8** |
| **ALLANTOIN** | **0.1** | **ALLANTOIN** | **0.1** |
| **DIPOTASSIUM GLYCYRRHIZATE** | **0.1** | **SACCHAROMYCES FERMENT** | **2** |
| **DIMETHICONE** | **2** | **BACILLUS FERMENT** |  |
| **CETEARYL DIMETHICONE CROSSPOLYMER** |  | **SCUTELLARIA BAICALENSIS ROOT EXTRACT** |  |
| **1,2-HEXANEDIOL** | **0.5** | **CYCLODEXTRIN** |  |
| **PENTAERYTHRITYL TETRA-DI-t-BUTYL HYDROXYHYDROCINNAMATE** | **0.02** | **SODIUM CHLORIDE** |  |
| **CETEARYL ALCOHOL** | **2** | **DIPOTASSIUM GLYCYRRHIZATE** | **0.1** |
| **CETYL PALMITATE** | **0.5** | **DIMETHICONE** | **2** |
| **SORBITAN PALMITATE** |  | **CETEARYL DIMETHICONE CROSSPOLYMER** |  |
| **SORBITAN OLIVATE** |  | **1,2-HEXANEDIOL** | **0.5** |
| **CETEARYL OLIVATE** | **2.5** | **PENTAERYTHRITYL TETRA-DI-t-BUTYL HYDROXYHYDROCINNAMATE** | **0.02** |
| **SORBITAN OLIVATE** |  | **CETEARYL ALCOHOL** | **2** |
| **AMMONIUM ACRYLOYLDIMETHYLTAURATE/VP COPOLYMER** | **0.2** | **CETYL PALMITATE** | **0.5** |
| **ARGININE** | **0.23** | **SORBITAN PALMITATE** |  |
| **POLYMETHYLSILSESQUIOXANE** | **0.5** | **SORBITAN OLIVATE** |  |
|  |  | **CETEARYL OLIVATE** | **2.5** |
|  |  | **SORBITAN OLIVATE** |  |
|  |  | **AMMONIUM ACRYLOYLDIMETHYLTAURATE/VP COPOLYMER** | **0.2** |
|  |  | **ARGININE** | **0.23** |
|  |  | **POLYMETHYLSILSESQUIOXANE** | **0.5** |
